# The Assessment of the Rank of Torque Control during Incisor Retraction and Its Impact on the Resorption of Maxillary Central Incisor Roots According to Incisive Canal Anatomy—Systematic Review

**DOI:** 10.3390/jcm12082774

**Published:** 2023-04-08

**Authors:** Anna Ewa Kuc, Jacek Kotuła, Jakub Nawrocki, Alicja Babczyńska, Joanna Lis, Beata Kawala, Michał Sarul

**Affiliations:** 1 Department of Dentofacial Orthopedics and Orthodontics, Wroclaw Medical University, Krakowska 26, 50-425 Wroclaw, Poland; 2 Dental Star Specialist Aesthetic Dentistry Center, 15-215 Białystok, Poland; 3 Department of Integrated Dentistry, Wroclaw Medical University, 50-425 Wroclaw, Poland

**Keywords:** resorption, incisive canal, nasopalatine canal, retraction

## Abstract

Background: Root resorption is one of the complications of orthodontic treatment, and has a varied and unclear aetiology. Objective: To evaluate the relationship between upper incisor resorption and contact with the incisive canal and the risk of resorption during orthodontic treatment associated with upper incisor retraction and torque control. Search methods: According to PRISMA guidelines, the main research question was defined in PICO. Scientific databases MEDLINE, EMBASE and the Cochrane Central Register of Controlled Trials were searched for linking keywords: Resorption of roots incisive canal, Resorption of roots nasopalatine canal, Incisive canal retraction and Nasopalatine canal retraction. Selection criteria: No time filters were applied due to the significantly limited number of studies. Publications in the English language were selected. Based on the information provided in the abstracts, articles were selected according to the following criteria: controlled clinical prospective trials and case reports. No randomised clinical trials (RCTs) or controlled clinical prospective trials (CCTs) were found. Articles unrelated to the topic of the planned study were excluded. The literature was reviewed, and the following journals were searched: American Journal of Orthodontics and Dentofacial Orthopedics, International Orthodontics, Journal of Clinical Orthodontics, Angle Orthodontist, Progress in Orthodontics, Orthodontics and Craniofacial Research, Journal of Orofacial Orthopedics, European Journal of Orthodontics and Korean Journal of Orthodontics. Data collection and analysis: The articles were subjected to risk of bias and quality assessment using the ROBINS-I tool. Results: Four articles with a total of 164 participants were selected. In all studies, differences in root length were observed after contact with the incisive canal, which was statistically significant. Conclusions and implications: The contact of incisor roots with the incisive canal increases the risk of resorption of these roots. IC anatomy should be considered in orthodontic diagnosis using 3D imaging. The risk of resorption complications can be reduced by appropriate planning of the movement and extent of the incisor roots (torque control) and the possible use of incisor brackets with built-in greater angulation. Registration CRD42022354125.

## 1. Introduction

Planning orthodontic treatment often involves the extraction of the first premolars. In malocclusions associated with protrusion and in cases of high skeletal discrepancy, maximum incisor retraction is often necessary to improve not only occlusion, lip position and the patient’s facial and smile profile, but also periodontium protection [1,2,3]. The introduction of orthodontic mini-implants as a maximum anchorage has enabled much more effective treatment and greater tooth displacement. However, the limit of maximum incisor retraction has been debatable for years. The accepted standard, according to the envelope of discrepancy established by Profitt and Ackerman in 1994, is the possibility of the retraction of the upper incisors by approximately 7 mm [4]. The determination of these dimensions was based on 2D radiographs and the presence of the cortical plate. In the era of widespread availability of CBCT examination with the possibility of using TISAD, the anatomy of each patient can be carefully and individually analysed, with retraction in excess of 7 mm being a possibility for application [5]. The use of retraction is characterised by longer treatment times, the use of greater forces and tooth movement over a greater distance compared to other types of treatment. The above characteristics may be the causes of orthodontically induced inflammatory root resorption (OIIRR) [6,7,8,9,10,11,12,13,14,15]. Tooth root resorption during orthodontic treatment is one of the most common iatrogenic complications [6]. Many factors contribute to this phenomenon. In recent years, as a result of the development of 3D imaging, attention has been drawn to another important element, namely the incisive canal, and its relationship to upper incisor roots. The incisive canal, also known as the nasopalatine canal, is the connection between the nasal cavity and the oral cavity, containing vessels and nerves within it. It is an often overlooked element in the orthodontic treatment planning process, but it is surrounded by a relatively thick cortical plate. As there is evidence of an effect of the buccal and palatal cortical plate on the induction of root resorption [6,16,17], the cortical plate of the incisive canal could be an analogous factor. In this systematic review, the authors attempted to gather evidence on the relationship between resorption and the presence and anatomy of the incisive canal, as well as the circumstances under which the canal collides with roots, and also to assess the risk in individual patient groups during orthodontic treatment and the role of incisor torque control depending on growth direction, skeletal class, gender or treatment with or without incisor retraction.

### Objective

The aim of the study was to analyse the possibility of minimizing the resorption of incisor roots during retraction through appropriate treatment planning and control of their inclination in relation to the individual anatomy of the incisive canal.

## 2. Materials and Methods

### 2.1. Protocol and Registration

The systematic review was registered in the PROSPERO database under the identification number CRD42022354125. The study was conducted in accordance with PRISMA guidelines [18]. Due to the type of the study, there was no patient participation, no intervention, and no requirement to collect any personal data, hence ethical approval was not requested.

### 2.2. Eligibility Criteria

The study design was defined in PICO format: Population (P)—patients with complete permanent dentition; Intervention (I)—orthodontic extraction treatment with braces using a straight wire technique with incisor retraction; Comparison (C)—assessment of the distance between the upper incisor roots and the canal before and after treatment and assessment of the length of the incisor roots before and after treatment; Outcome (O)—statistically significant/non-significant differences in the distance between the upper incisor roots and the canal before and after treatment and in the length of the incisor roots before and after treatment.

Due to the significantly limited number of studies, randomised clinical trials (RCTs), controlled clinical prospective trials (CCTs), systematic reviews, retrospective studies and case reports were included. Only publications in the English language were selected.

### 2.3. Information Sources and Search Strategy

The authors (A.E.K., J.K. and J.N.) conducted an independent search of the following electronic databases: PubMed, EMBASE and the Cochrane Central Register of Controlled Trials, Web of Science, Scopus, by entering the following keywords:

- Root resorption incisive canal;
- Root resorption nasopalatine canal;
- Incisive canal retraction;
- Nasopalatine canal retraction.

The literature was reviewed, and the following journals were manually searched: American Journal of Orthodontics and Dentofacial Orthopedics, International Orthodontics, Journal of Clinical Orthodontics, Angle Orthodontist, Progress in Orthodontics, Orthodontics and Craniofacial Research, Journal of Orofacial Orthopedics, European Journal of Orthodontics and Korean Journal of Orthodontics. Handsearching was performed by screening similar articles under every article found by keywords. No time filters or status were applied. All the databases were searched from 14 July 2022 to 31 July 2022. Grey literature sources were screened, such as Pro-Quest Dissertations and Theses Global and Google scholar. If data from the study reports were insufficient, unclear, or missing, we attempted to contact the study authors for additional information. If we judged that the missing data might render the result uninterpretable, we excluded the data from the analysis and clearly stated the reason.

### 2.4. Study Selection

The authors (A.E.K., J.K. and J.N.) independently searched databases and, after duplicate removal, reviewed titles by their relevance to the topic of this systematic review. Articles included after title screening were evaluated thoroughly. Due to the limited number of on-topic studies, no exclusion criteria were applied. The reviewers were blinded to each other’s decisions. The authors discussed any disagreements until a consensus was reached, and if necessary the fourth author (MS) was consulted.

### 2.5. Data Collection and Data Items

The following data were extracted to Microsoft Excel: sample size, year of publication, author’s name, mean amount of root resorption after intervention, difference between mean root resorption in control and retraction group, standard deviation for listed data, general characteristics of each group, general characteristics about intervention associated with each group. Study investigators (authors of the articles accepted in the systematic review) would be contacted for unreported data or additional details.

### 2.6. Risk of Bias in Individual Studies

In accordance with the Cochrane Handbook for Systematic Reviews of Interventions, the Risk of Bias (RoB) was achieved using Risk of Bias In Non-randomized Studies of Interventions (ROBINS-I tool) [19]. It was planned to use the Cochrane risk-of-bias tool (RoB 2) for randomized trials; however, due to the absence of RCTs, using the RoB 2 tool was unnecessary. An overall judgement about the risk of bias was reached after completing 7 main domains for each study. The outcome of overall bias could be: 1. low risk of bias, 2. moderate risk of bias, 3. serious risk of bias, 4. critical risk of bias, 5. no information. The evaluation was performed by 2 authors (A.E.K. and J.N.) independently. The authors discussed any disagreements until a consensus was reached, and if necessary the last author (MS) was consulted.

### 2.7. Summary Measures, Synthesis of Results and Additional Analyses

The planned formal method of combining individual study data, randomised and controlled clinical studies was statistically evaluated both jointly (by heterogeneity analysis—the Cochrane Q test and I² statistics, and random-effect meta-analysis) and separately (statistical importance between groups in each study) with subgroup analysis and significance established at *p* < 0.05. Results of the analyses will be presented graphically with forest plots after comparisons of study designs, methodologies and participants, to judge the clinical heterogeneity of the studies. Unfortunately, due to the lack of RCTs and CCTs and an inability to test the heterogeneity of the studies, this prevents the conducting of statistical analysis and meta-analysis.

## 3. Results

**Figure 1:**
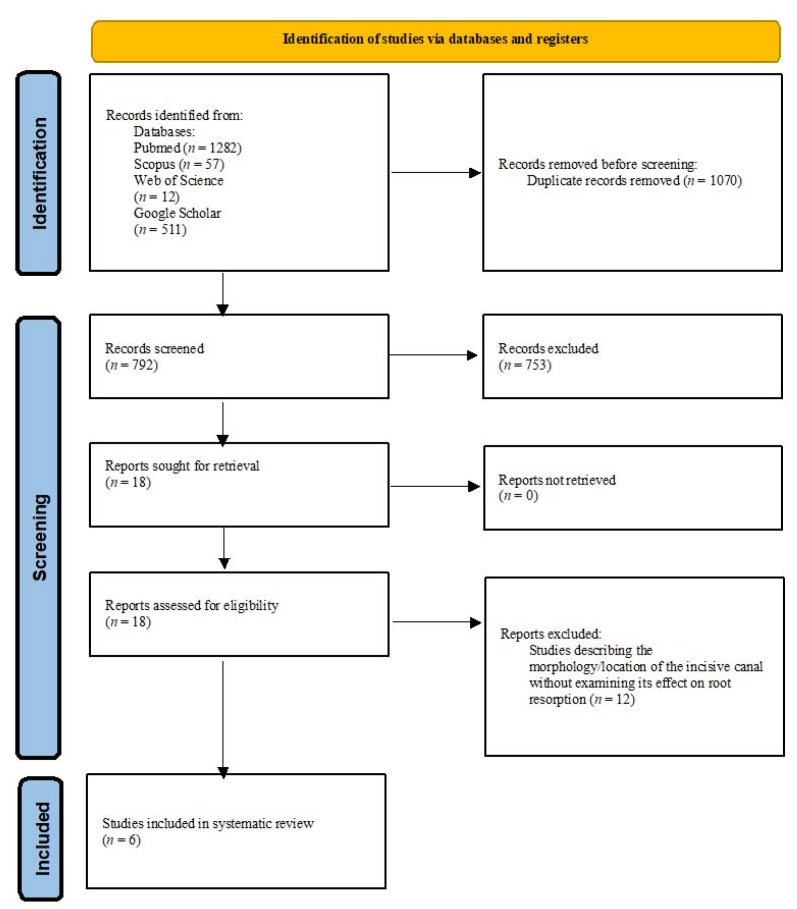
PRISMA flow diagram of the literature selection process.

The keywords yielded 1862 abstracts. Thirty-nine articles were initially validated as eligible for the systematic review, and they were analysed in detail. In the end, six articles were selected, including four controlled clinical prospective trials and two case reports. The full selection process is shown in Figure 1.

### 3.1. Group Population

The total number of participants was 164. The average group population was 33 patients. The largest group was found in the articles by Yu et al. [1], with 35 participants in the group. The smallest group was included in the study by Nakada et al. [20], with 30 participants. Only one study included a control and a retraction group [18]; in the remaining studies, only retraction groups were present (Table 1).

### 3.2. Age and Gender

In most studies, participants were adult patients; in the study by Pan et al. [2], participants were also adolescents. In each study, the female group population was larger than the male group population; see Table 1.

### 3.3. Treatment Strategy

In treated patients, extractions of maxillary premolars were performed to gain space for incisor and canine retraction. In two studies, maximum anchorage in the form of TISAD was applied to the study groups. In the remaining two groups, there was no information on the use of TISAD (Table 1).

### 3.4. Risk Analysis

The main parameters were a change in the length of the incisor roots before and after treatment and the distance between the roots and the incisive canal before and after treatment.

In all studies, a relationship was observed between the resorption of the upper incisor roots and their proximity to the incisive canal.

In all studies, the root resorption of the central upper incisors occurred during incisor retraction. The greatest resorption was observed in the study by Chung, in a group with canal invasion and without remodelling 3.3 ± 1.54 mm (Table 1).

### 3.5. Changes in the Length of the Central Incisor Roots in Contact with the Incisive Canal

In all articles, the shortening of the upper incisor roots after retraction was statistically greater when contact was made with the incisive canal. The results of statistically significant studies are presented below (Table 1).

Yu et al. [1] revealed a greater root resorption in the retraction group (2.3 ± 1.40 mm) compared to the control group (1.1 ± 0.75 mm). In addition, subgroups were created according to the distance from the roots to the IC after treatment (separation, approximation, contact, invasion). The closer the incisor root was to the incisive canal, the greater the resorption, but this result was not statistically significant. The retraction group was four times more likely to have root invasion or contact with the incisive canal compared to the control group. In 11.4 percent of the retraction group, there was a change in the course of the incisive canal, which may point to its remodelling ability.

Chung et al. [3] found that 53 percent of retraction treatment cases resulted in the invasion of the incisive canal by the incisor roots. A higher risk of contact between the roots and canal was shown when the inter-root distance was less than the width of the incisive canal. In the subgroup with invasion, resorption was statistically higher (2.4 ± 1.59 mm) than in the non-invasive group (0.8 ± 0.96 mm) *p* < 0.0001. Resorption was lower in patients with invasion and remodelling (1 ± 0.92 mm) compared to patients without remodelling (3.3 ± 1.54 mm) *p* < 0.0001.

Pan et al. [2] showed a significantly greater shortening (2.63 ± 0.93) of the incisor roots in the contact group between the roots and the incisive canal than in the group without contact (1.14 ± 0.83), possibly pointing to their positive correlation. With uncontrolled tipping of the incisors, there is an increased risk of canal invasion in the cervical area. The risk of root contact is increased when the position of the incisive canal is low.

Nakada et al. [20] showed that root resorption was statistically greater on the side closer to the incisive canal than on the opposite side. In addition, the range of palatal resorption on the side closer (2.49 ± 0.61) to the canal was also greater than on the farther side (1.51 ± 0.49 mm). Ultimately, he concluded that the proximity of the apex to the IC cortical plate was a factor in root resorption.

Imamura et al. [22] described a clinical case of a patient in whom, after the retraction of the incisors, the root of the one in contact with the incisive canal resorbed at the contact area (3.6 mm). Therefore, the size and morphology of the IC was considered to have an impact on root resorption.

Chung et al. [21] also described a case of root resorption after contact with the IC, together with tooth vitality preservation.

### 3.6. Risk of Bias

**Figure 2:**
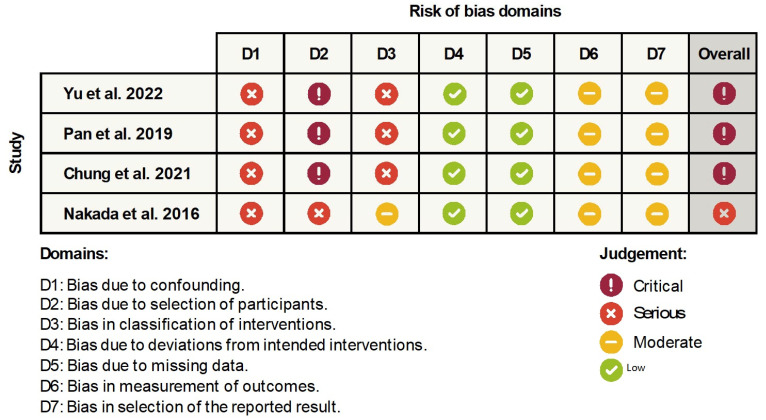
The results of the bias risk assessment according to the ROBINS-I tool [1,2,3,20].

The risk of bias analysed according to the ROBINS-I tool (Figure 2) can be described as critical for all articles but one—Nakada et al. [20], where the risk of bias was serious. Despite the critical and serious overall risk of bias, the studies may be accepted as useful. Only one domain categorized studies at high or critical risk, and it was caused by the nature of the research. In the study by Pan et al., retraction was performed using TISAD (maximum anchorage), but there is no information on whether the placement of the mini-implants was the same in all cases and thus whether the force vector followed a similar course. The researchers statistically analysed the retraction distance and the difference in incisor inclination before and after retraction, but did not tabulate specific values. Similarly, there was no information on the type of brackets or whether each patient was treated with the same prescription. In the study by Yu et al., the extent of retraction (determinant of group membership—<2 mm control, >4 mm retraction) was given, but there was no information on the method of treatment other than that the retraction group involved premolar extraction. In addition, there was no information about torque control during treatment and no information about the type of brackets and whether each patient in the control and retraction groups was treated in the same way. The study by Nakada et al. only considered one group out of all those studied—only in this group could there be an association between the presence of the incisive canal and root resorption. There was no information about the method of treatment, extent of retraction or torque control; only information on the distance by which the central incisors were moved was provided. The small sample size is also a factor of error. The best-matched study group was represented by the study by Chung et al. [3]. It reported the average treatment time, and the patients qualified for the study met certain conditions: Skeletal Class I or II malocclusion, bimaxillary protrusion, treatment completed with Class I canine relationship and retraction >4 mm. Study group exclusion criteria were investigated and TISAD was used, but there was no information on its location or type of brackets, torque control or retraction distance (the only information was >4 mm).

### 3.7. Analysis of Results

The planning of orthodontic treatment of malocclusions associated with bimaxillary protrusion and a skeletal Class 2 malocclusion with incisor protrusion often involves premolar tooth extraction to make room for maximum incisor retraction. Orthodontic mini-implants, which have been in use for many years, enable maximum anchorage and a range of retraction that can exceed the patient’s anatomical conditions. Often, 7 mm in the maxilla, as determined by Profitt and Ackerman, is given as the maximum retraction range based on the palatal cortical plate, which is a limiting factor [4,23]. The studies analysed by the authors on the change in the incisor root length after contact with the incisive canal and the sheer diversity of the morphology of the incisive canal in terms of possible contact with the incisor roots show that this range may be smaller in some patients because it is located between the roots of the incisor teeth and the palatal cortical plate, and may be the first to stand in the way of the displaced maxillary incisors. As is common knowledge, the incisive canal, also known as the nasopalatine canal, is an anatomical structure located in the midline protecting the incisive nerve and blood vessels [24,25]. The introduction of CBCT imaging enabled a more accurate diagnosis and analysis of anatomical structures in the field of orthodontic displacement, with these images providing more complete information compared to panoramic radiography and lateral cephalometric radiography [25]. There are few studies or publications on the possible relationship between canal morphology and proximity to incisor roots in terms of orthodontic treatment complications. However, the current publications included in this systematic review clearly indicate a possible higher risk of resorption of the incisor roots during retraction and lateral displacement or intrusion after contact with the cortical plate of the incisive canal [1,2,3,20]. These studies are characterised by moderate evidential value due to the nature of controlled clinical prospective trials. Among these, Chung’s study [3] shows the highest evidential value due to the lowest risk of bias.

## 4. Discussion

The pathomechanism of root resorption during orthodontic treatment is the effect of the damage of cementoblasts and precement, as well as an imbalance between the resorption effect of osteoclasts and the apposition effect of cementoblasts during the action of a stimulating factor. A symptom of root resorption is a change in shape and shortening of tooth roots to varying degrees [26,27,28]. In order to inhibit resorption, the stimulator must stop working. In orthodontic treatment, this means disabling any orthodontic forces to allow the osteoblasts to rebuild lost tissue. In the case of first and second degrees of resorption, the shape of the apex changes, while in the case of circular apical resorption (third degree), the length of the root is irreversibly shortened [26,27,28].

In addition, according to the latest knowledge, photomodulations such as low-level laser therapy (LLLT), light-emitting diodes (LED) and low intensity pulsed ultrasound (LIPUS) can have a positive effect on the average total root resorption [29].

**Figure 3:**
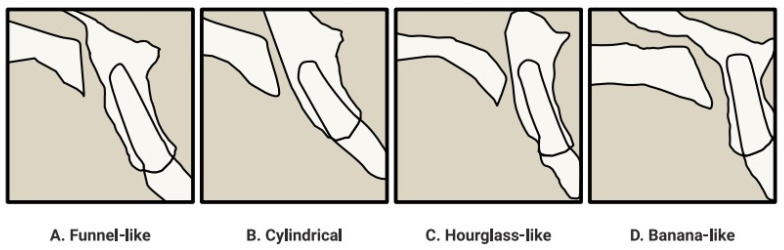
Variety of shapes and courses of the incisive canal [1].

Incisor resorption may be associated with incisor retraction. Important factors include the extent of retraction and the degree of torque control. The studies reviewed show that the extent of retraction is individualised and strictly dependent on the patient’s anatomical structures, while the degree of torque control is important during retraction because of the ability to assess the degree of root displacement in the maxillary structure. The analysis of CT scans of different patients pointed to the existence of four main shapes of the incisive canal, listed according to their frequency [23]: funnel-shaped, cylindrical-shaped, hourglass-shaped and banana-shaped (Figure 3). This canal can be straight (<10 deg. to the plane of the palate), slanted (>10 deg. to the plane of the palate) and may also be characterised by additional curvature [1].

According to the study by Arnaut, whose results overlapped with those of Milanovic and Thakur, the shape of the incisive canal itself has no relation to gender [23,30,31]. The average canal length obtained by Arnaut et al. and Bornstein et al. is slightly over 10 mm [23,32]. Meanwhile, the analysis of axial CBCT sections provided information that the average width of the incisive canal was 3.59 mm [23,31,33]. Importantly, according to the study by Cho et al., the incisive canal was wider than the inter-root distance in more than 60 percent of the patients [34]. The average anterior-posterior distance between the central incisors roots in the maxilla and the incisive canal is 5–6 mm [34,35]. The detailed results of the study by Arnaut et al. on the morphology and shape of the IC also showed that the diameter of the incisive canal depends on shape and was significantly increased in those with a banana shape, and decreased in those with a cylindrical shape [23]. This suggests that patients with the banana-shaped incisive canal are more prone to contact between the roots and canal during retraction than others. An increase in the distance between the roots and the incisive canal in the apical direction was also observed. Therefore, in the era of 3D imaging, it is worthwhile to individually plan the anatomically possible extent of retraction and case-appropriate torque control during retraction, and perhaps to use brackets with built-in greater angulation for maxillary incisors to increase the interapical distance and thus allow the roots to bypass the IC [23,34,36,37]. A reduction in the AP NF dimension in the case of the banana shape is a limiting factor for tooth retraction. At the same time, a cylindrical-shaped canal is accompanied by a higher risk of root invasion after retraction due to a significant reduction in the space required for retraction movement [23].

**Figure 4:**
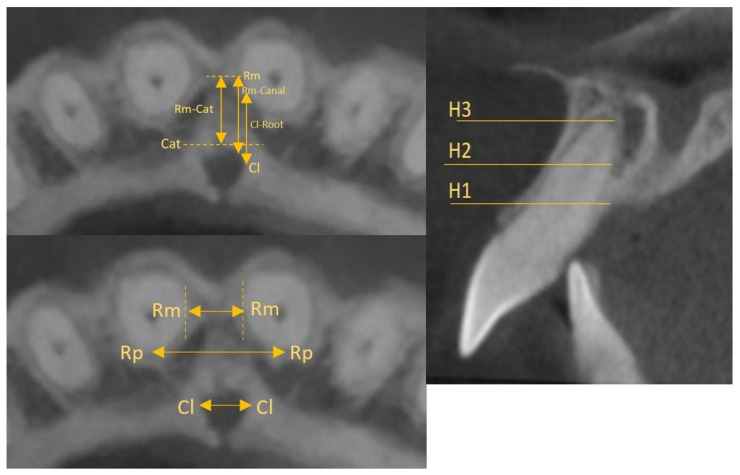
Topography and linear measurements [35].

On the other hand, the analysis by Al-Rokhami et al. on the relationship of canal morphology with growth direction, skeletal class and gender showed that women with an increased vertical jaw relation are more likely to have contact between the roots and the incisive canal due to the width of the canal being greater than the inter-root distance, especially at the level of the incisive foramen H2, i.e., half the distance between the root apex and the lowest point of the incisive foramen on the buccal wall [35] (Figure 4). In patients with an incisive canal wider than the inter-root distance, it is worth considering the use of brackets with integrated greater angulation on the maxillary incisors. The retraction of incisors after previous maxillary expansion with palatal suture expansion or maxillary distraction osteogenesis may require special care and careful diagnosis due to the concomitant widening of the incisive canal—a subject worthy of attention and careful research. Furthermore, it was shown that the incisive canal is closer to the roots in high-angle patients than in medium- and low-angle patients. In addition, men are characterised by greater sagittal distances of incisor roots to the canal compared to women. The analysis of the above information suggests that high-angle women are at greater risk of contact with the incisive canal.

According to the study by Arnaut, whose results overlapped with those of Milanovic and Thakur, the shape of the incisive canal itself has no relation to gender [23,30,31]. The average canal length obtained by Arnaut et al. and Bornstein et al. was slightly over 10 mm [23,32]. Meanwhile, the analysis of axial CBCT sections provided information that the average width of the incisive canal was 3.59 mm [23,31,33]. Importantly, according to the study by Cho et al., the incisive canal was wider than the inter-root distance in more than 60 percent of the patients [34]. The average anterior–posterior distance between the central incisors’ roots in the maxilla and the incisive canal was 5–6 mm [34,35]. The detailed results of the study by Arnaut et al. on the morphology and shape of the IC also showed that the diameter of the incisive canal depended on shape and was significantly increased in those with a banana shape, and decreased in those with a cylindrical shape [23]. This suggested that patients with the banana-shaped incisive canal are more prone to contact between the roots and canal during retraction than others. An increase in the distance between the roots and the incisive canal in the apical direction was also observed. Therefore, in the era of 3D imaging, it is worthwhile to individually plan the anatomically possible extent of retraction and case-appropriate torque control during retraction, and perhaps to use brackets with built-in greater angulation for maxillary incisors to increase the interapical distance and thus allow the roots to bypass the IC [23,34,36,37]. A reduction in the AP NF dimension in the case of the banana shape is a limiting factor for tooth retraction. At the same time, a cylindrical-shaped canal is accompanied by a higher risk of root invasion after retraction due to a significant reduction in the space required for retraction movement [23].

Additional data on contact risk were presented in the study by Costa et al. Their results showed that low-angle patients have a thicker alveolar bone in the maxillary anterior area, which translates into greater distances between the roots and the incisive canal. They found no relationship between the facial profile and canal volume. However, they noted that men had a wider canal than women regardless of growth direction [38]. Canal height did not differ significantly between adults and adolescents, but was significantly lower for patients with contact after retraction, suggesting that the low position of the incisive canal may be a risk factor.

In the studies by Matsumura et al. and Linjawi et al., a significantly positive relation-ship was noted for the angles between the incisive canal and the palatal plane as well as between the long axis of the central incisors and the palatal plane—the more tilted the incisors, the more slanted the incisive canal [36,39]. Consequently, orthodontic treatment involving incisor tilting and retraction increased the risk of contact in these patients, mainly in the lower root half and cervical area. Thus, analysing the above, it can be concluded that excessive tilting may favour cervical resorption, whereas excessive torque may favour apical resorption. The use of 3D imaging and the visualisation of the incisive canal drew the attention of researchers to the importance of its morphology, shape, course and the possible relationship with incisor root resorption occurring after retraction. The amount of studies on the change in incisor root length due to contact with the IC is fairly limited, and all of them are retrospective. In this systematic review, all of them showed a positive relationship between the degree of resorption and the reduction in the distance from the roots to the IC. In addition, the study by Yu et al. [1] demonstrated the possibility of remodelling the canal in response to orthodontic tooth movement, which was accompanied by a lower degree of resorption. In 11.4 percent of the patients, a change in canal direction from slanted–straight to slanted–curved was observed after retraction. Further high-quality studies are needed that would perhaps make the remodelling ability of the IC dependent on the force applied, the tooth displacement times or other orthodontic factors, possibly reducing the number of root resorption complications. A statistically significant difference in the length of the roots not having and having contact with the incisive canal after orthodontic treatment with incisor retraction has been proven in controlled clinical prospective trials included in this systematic review with a moderate risk of bias.

## 5. Limitations

The main limitations of the review include articles written in English, which may affect the risk of bias of this publication. Furthermore, the amount of studies analysing the topic addressed was considerably limited. Controlled clinical retrospective trials and case reports were analysed due to the lack of randomised clinical trials and controlled clinical prospective trials. The inability to test the heterogeneity of the studies prevents the conduction of a meta-analysis.

## 6. Conclusions

The studies showed that contact between the upper incisor roots and the incisive canal significantly increased the risk of resorption of these roots. The diversity of the morphology of the incisive canal and its relationship to incisor roots suggests the need for more accurate diagnosis using 3D imaging, and the extent of possible retraction without a high risk of resorption and individualised control of incisor torque during retraction.

The incisive canal may have a remodelling ability in response to orthodontic displacement—more thorough research is needed to show this ability as dependent on factors such as age, gender or applied force.

Further high-quality studies—primarily RCTs with clearly defined methodology—are needed for better quality analyses and more reliable conclusions.

## Figures and Tables

**Table 1:** Studies included in the Systematic Review.

Reference	Patients	Groups	Age (Years Old)	Patients Malocclusion	Treatment Method	Assessment Method	Results
Yu et al., 2022 [1]	*n* = 67 (M20 F47)	G1 = 32 G2 = 35 G1-control G2- retraction	Mean ± SD G1 = 25.9 ± 5.85 G2 = 26.2 ± 8.34	Skeletal class I or class II (0° < ANB < 7°)	G1-without pre-molar extractions, <2 mm horizontal maxillary incisor tip movement G2- with pre-molar extractions, >4 mm retraction of the maxillary retraction	Pre- and post-treatment CBCT	Control = root resorption = 1.1 ± 0.75 mm; Retraction group 2.3 ± 1.40
Nakada et al., 2016 [20]	*n* = 30 (M8 F22)	G0 = G1 + G2 + G3 = 30 G1 (Labial movement) = 9 G2 (Palatal movement) = 16 G3 (Midline shift) = 5	Mean ± SD 21.92 ± 5.83	n/a	G0 patients who underwent four-bicuspid extraction followed by treatment with multibracket appliances G1 (Labial movement) G2 (Palatal movement) G3 (Midline shift)	Pre- and post-treatment CBCT	Apical root resorption: Mean ± SD = 1.80 ± 0.82 mm Maximum = 3.96 mm; Minimum = 0.18 mm; Resorption in MS group: mesial mean ± SD = 2.49 ± 0.61; distal mean ± SD = 1.51 ± 0.49 mm
Pan et al., 2019 [2]	*n* = 33 (M10 F23)	G1 = 33	Adult (*n* = 20): Mean ± SD = 25.35 ± 5.12; Teenager (*n* = 13): Mean ± SD = 12.76 ± 1.09	Skeletal class I or class II or molar relationship, Convex profile	Extraction of at least two upper premolars; Retraction of the upper anterior teeth with mini-implants (maximum retraction)	Pre- and post-treatment CBCT	Root Length Decrease Noncontact group 1.14 ± 0.83 Contact group 2.63 ± 0.93, *p* value **≤** 0.01
Chung et al., 2015 [21]	*n* = 2 (F2)	G1 = 2	Age, years: 19.46	Skeletal class II malocclusion with a protrusive profile	Extraction of 4 first premolars, retraction using TADs (maximum retraction)	Pre- and post-treatment CBCT and panoramic radiograph	Patient 1 “severe apical root resorption”
Chung et al., 2021 [3]	*n* = 34 (M8 F26)	G1 = 34 retraction >4 mm	Mean ± SD 26.7 ± 8.8	Skeletal class I or class II (0° < ANB < 7°) bimaxillary protrusion	>4 mm retraction of the upper incisor (U1) using TADs (maximum retraction)	Pre- and post-treatment CBCT	Classification (N), Non-invasion (N = 32) Mean ± SD = 0.8 ± 0.96 mm Invasion (N = 36) Mean ± SD = 2.4 ± 1.59 mm IC remodelling (+) (N = 18) Mean ± SD = 3.3 ± 1.54 mm IC remodelling (−) (N = 18) Mean ± SD = 1 ± 0.92 mm
Imamura et al., 2020 [22]	*n* = 1 (F1)	G1 = 1	Age, years: 21	Angle class I molar, class II canine relationships, overjet 5.3 mm, overbite 6.1 mm, linguoversion of the maxillary incisor (crossbite), Discrepancies = −9.4 mm (maxilla), −5.5 mm (mandible)	Extraction of the bilateral maxillary first premolars and mandible second premolars, TADs in the maxilla (maximum retraction)	Pre- and post-treatment CBCT	Displacement of the roots, right = 2.3 mm; left = 3.1 mm Resorption, right = 3.6 mm; left = 1.8 mm

Abbreviations: F, female; M, male; G0, group 0; G1, group 1; G2 group 2, G3, group 3; ANB, angle formed by point A, nasion (N) and point B; CBCT, cone beam computed tomography; n/a, not applicable.

## Data Availability

The data sets that were used and/or analysed in this study are available from the corresponding author upon reasonable request.

## Abbreviations

The following abbreviations are used in this manuscript:

PRISMA	Preferred Reporting Items for Systematic Reviews and Meta-Analyses
PICO	Population, Intervention, Comparison, Outcome
RCT	Randomised Clinical Trial
CCT	Controlled Clinical Prospective Trial
ROBINS-I	Risk of Bias In Non-Randomised Studies of Interventions
CBCT	Cone Beam Computed Tomography
TISAD	Temporary Intraoral Skeletal Anchorage Device (orthodontic mini-implant)
